# A systematic literature review and case study on the social impact of the other women’s contributions to education and dialogic feminism

**DOI:** 10.3389/fsoc.2024.1477983

**Published:** 2024-12-09

**Authors:** Laura Ruiz-Eugenio, Rosa Valls-Carol, Ainhoa Flecha, Adriana Aubert

**Affiliations:** ^1^Department of Theory and History of Education, University of Barcelona, Barcelona, Spain; ^2^Department of Sociology, Autonomous University of Barcelona, Bellaterra, Spain; ^3^Department of Sociology, University of Barcelona, Barcelona, Spain

**Keywords:** dialogic feminism, other women, adult education, social impact, Schools as Learning Communities, Dialogic Gatherings, successful educational actions, sociology

## Abstract

Feminism has been one of the most important social movements of the last centuries. Current societies have widely recognized their contributions. However, whereas ethnic diversity has been included in the movement, diversity in terms of academic background has not. Therefore, the contributions made by the “other women” from their daily lives, those with no university education (who belong to cultural minorities or not), remain on the margins of public debate. In the face of this reality, the plurality of all women has been building contributions to education and dialogic feminism. Based on Lidia Puigvert’s previous study on dialogic feminism and the “other women” movement, this article aimed to trace the social impact of “other women’s” contributions to education and dialogic feminism in their lives and communities. A qualitative case study has been developed that collects a systematic literature review, in-depth interviews with educators, focus groups, and communicative daily life stories with women who have participated in Schools as Learning Communities and democratic adult education associations in Spain over the last 20 years. The results provided evidence about the contributions of the “other women” in the development and expansion of successful educational actions that have generated social and educational transformations in themselves, their families, and their communities, such as Dialogic Gatherings and the prevention of gender violence, among others.

## 1 Introduction

The analysis of the social impact of the contributions of “other women” to education and dialogic feminism is one of the case studies carried out in the framework of the ALL WOMEN research project (2021–2024), funded by the State Research Agency of the Ministry of Science and Innovation of the Government of Spain. To create knowledge to advance SDG5 “Achieve gender equality and empower all women and girls,” the overall objective of the ALL WOMEN project is to identify actions in adult learning and education (formal and non-formal) that contribute to the empowerment of women who, because they do not have higher academic studies, have been left out of the spaces of debate and decision-making on issues that affect them directly and providing evidence of their social impact.

The study is framed within Lidia Puigvert’s conceptualization of dialogic feminism (Puigvert, 2001a,b). Puigvert was the first to theorize the inequality suffered by “other women” within the feminist movement. The “other women” are most women who, because they do not have a university degree, have been left out of the spaces of debate and decision-making in feminism. Many of them, in addition, suffer triple discrimination for being women, non-academic, and belonging to a cultural minority such as the Roma or being an immigrant. Puigvert (2001b) points out how many of these “other women” who participate in education come from these struggles carried out in the neighborhoods and villages from popular education projects. They have overcome enormous difficulties to study and, through their feminist liberation, contributing to refocusing feminism toward those women who suffer double or triple discrimination. Puigvert notes how these “other women” know it is difficult to mark the moment in history when they began their silent rebellion. However, they also know that the struggle against oppression and discrimination indeed has a history as long as that same oppression (Puigvert, 2001b, p. 29).

In 2001, Judith Butler, the most internationally referenced feminist, was invited to the “Women and Social Transformations” congress at the Barcelona Science Park. Butler shared a table with Lidia Puigvert, Elisabeth Beck-Gernsheim, and the “other women,” a cleaning worker participating in an adult school, a literate textile worker as an adult, and an illiterate Roma grandmother, among others. All of them were active participants in associations and projects for the right to quality education for all women and girls. Butler told Puigvert that it had been a beautiful and exciting experience that had changed her and her work and that it had given her back the most basic sense of why feminism is urgent, exciting, and creative (Puigvert and Flecha, 2010). That event helped to launch the foundations of dialogic feminism internationally since Puigvert (2001b) theorized it in her book *The Other Women* by collecting their contributions in a joint book by the three authors (Beck-Gernsheim et al., 2003).

Through this case study, evidence has been gathered of the social impact of the contributions of the “other women” to education and dialogic feminism from democratic adult education (AE) and its transfer to early childhood and primary education in the framework of Schools as Learning Communities. These contributions have improved women’s lives, families, and communities.

## 2 The study context

### 2.1 Democratic adult education movement and the “other women”

The “other women” in this case study are the participants in adult learning and educational actions in the framework of participants’ associations in the democratic adult education movement and Schools as Learning Communities (Flecha, 2000; Flecha and INCLUD-ED Consortium, 2015). Democratic education with a dialogic approach is based on Paulo Freire’s theory of dialogic action (Freire, 1970)and Ramon Flecha’s theory of dialogic society (Flecha, 2000, 2022), in which the participants, those who do not have higher academic degrees, are the protagonists of their own educational and transformation processes.

In Spain, toward the end of the 1960s and during the democratic transition in the 1970s, multiple experiences of dialogic popular education appeared in neighborhoods and towns. Some recover the dialogic cultural education promoted by the Free Women’s movement formed by working women before the Civil War of 1936 (Botton et al., 2005; Ruiz-Eugenio, 2011; Giner et al., 2016). One of these is the case of La Verneda-Sant Martí Adult School, created in 1978 in the neighborhood of Barcelona with the same name as the school (Giner, 2018). In 1996, FACEPA, Federation of Cultural and Educational Associations of Adults, was created in Catalonia to claim that non-academic participants have the capacity and the right to manage and decide on their own cultural and educational projects, as opposed to adult education, which turned participants into mere objects of the educators’ action (Oliver et al., 2009). Within this movement, the majority have always been women who, in the Spanish context of the post-war period and Franco’s dictatorship, had not had the opportunity to access education. Among these, toward the end of the 1990s and up to the present day, there are more and more immigrants from other countries, mainly Morocco (Botton et al., 2005).

Two main activities FACEPA organized were the Literacy Participants’ Congresses and the Dialogic Gatherings Congresses. In these congresses, the participants are the ones who make the interventions about their literacy projects or their experiences in the Dialogic Gatherings. In the Dialogic Gatherings, the best creations of humanity from literature, art, music, science, and mathematics are read and discussed. It is a collective construction of meaning and knowledge based on dialogue with all participants. This educational action was created in 1979 at the Adult Education School of La Verneda-Sant Martí and has been transferred all over the world to more than 15,000 preschools and primary and secondary schools, and to other types of institutions such as residential centers for minors, health centers, cultural associations, and prisons, among others (Ruiz-Eugenio et al., 2023b). The first congresses that took place during the first decade of the 2000s focused on Dialogic Literary Gatherings because they were the ones that spread the fastest. Most of the participants in these activities were women without basic academic qualifications. It is in this context and together with these women that Lidia Puigvert theorizes dialogic feminism. This is why most of the accounts in this case study include this profile of women.

### 2.2 Schools as Learning Communities

The Schools as Learning Communities project, inspired by the La Verneda-Sant Martí Adult School and promoted by the CREA Research Centre, was transferred to four primary schools in 1995 and would soon be replicated in many parts of the World (Roca et al., 2021). Today, it has become an international network that includes thousands of schools in various countries in Europe and Latin America that are implementing the successful educational actions (SEAs) that make up the project (SEAs4all Consortium, 2016; Step4Seas Consortium, 2017; ENLARGE Consortium, 2018; Natura Institute, 2019; Vieites et al., 2021). The SEAs are aimed at social and educational transformation based on dialogic learning, which aligns with international scientific theories highlighting two critical factors for learning today: interactions and community participation (Flecha, 2015). The SEAs are Interactive Groups, Dialogic Gatherings, family education, educative participation of the community, dialogic teacher training, and the dialogic model of conflict prevention and resolution. It is not the aim of this case study to explain each of them in detail, as there is abundant evidence that these SEAs obtain the most significant improvements in both instrumental learning and in values, emotions, and feelings at all ages and in a wide range of contexts (Morlà-Folch et al., 2022). Only those will be briefly described where evidence of the participation of the “other women” has been obtained from the literature review and the fieldwork carried out.

## 3 Methods

For the development of the case study, a systematic literature review on dialogic feminism and the participation of the “other women” in SEAs was carried out. Second, interviews with educators and teachers, daily life stories, and focus groups with a communicative approach were carried out with women participants in FACEPA associations and mothers from an Elementary School as a Learning Community in an urban context in a municipality of the Autonomous Community of Valencia. This federation has been selected because it brings together several associations and actively promotes the democratic adult education movement. In the case of the school, it was selected because of the project team members’ involvement in promoting SEAs, which also represents what happens in many other Schools as Learning Communities.

These specific criteria for the case study selection are part of the general selection criteria for ALL WOMEN research project case studies. These are as follows: 1. Existence of any previous evidence of social impact on achieving at least one target of UN goal 5, “Achieve gender equality and empower all women and girls”; 2. Include formal and non-formal adult learning and education programs; 3. Target low-skilled women or those without higher educational degrees.

### 3.1 Research question

What social impact (improvements for women themselves, their families, and communities) have the contributions of the “other women” to education and dialogic feminism had?

### 3.2 Development of the systematic literature review

A systematic literature review of articles published between 2001 and early 2024 in peer-reviewed journals on dialogic feminism and the participation of the “other women” in SEAs has been carried out, following the PRISMA standard criteria (Polanin et al., 2017; Tricco et al., 2018).

#### 3.2.1 Eligibility criteria, information sources, and search

**Figure 1:**
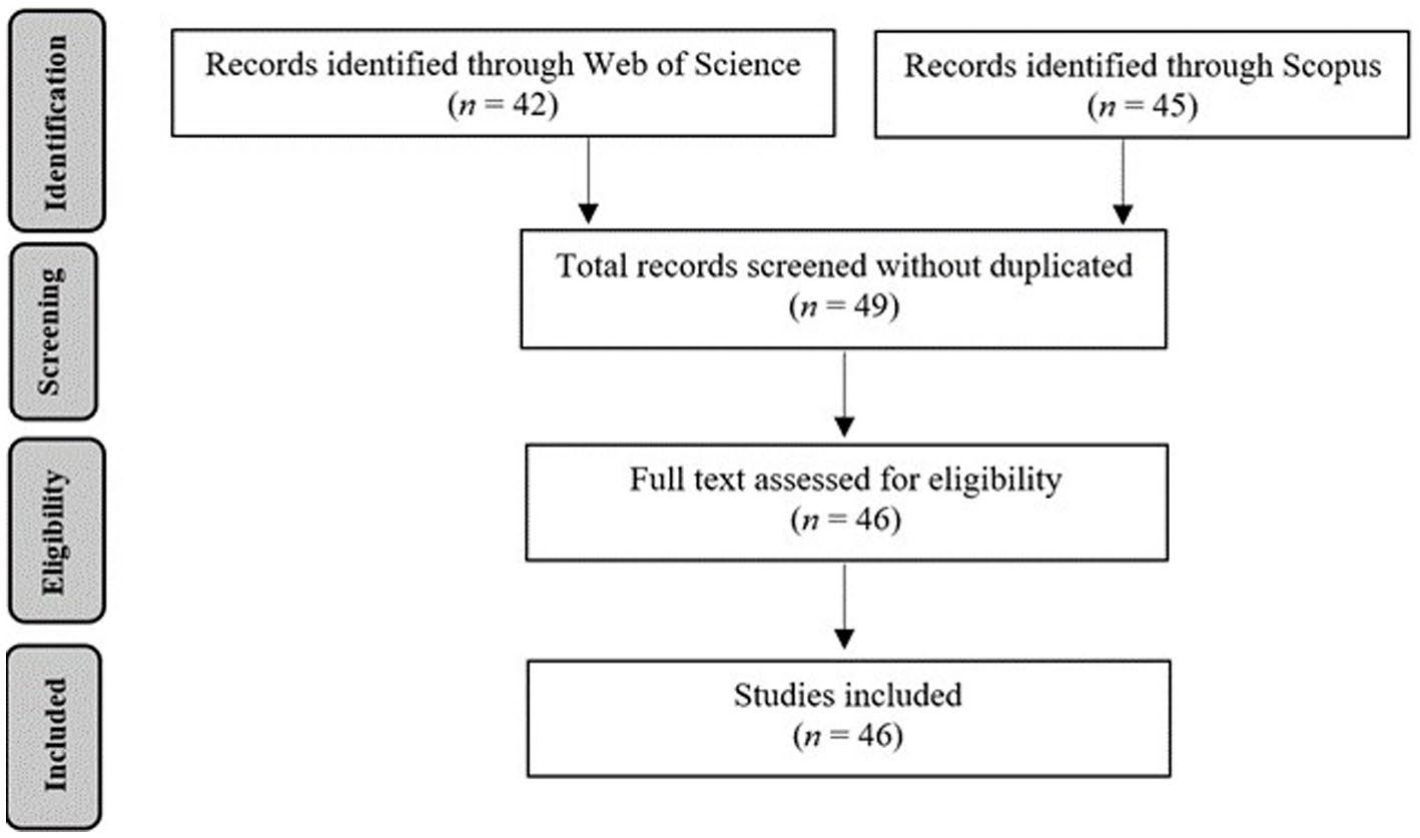
PRISMA flow diagram for search and screening process.

The eligibility of the articles responds to at least one of the following criteria: 1. They include Puigvert’s conceptualization of dialogic feminism; 2. They provide scientific evidence of the social impact of SEAs involving the “other women.” A total of 46 articles were selected. The search for articles was carried out in Web of Science (core collection), including articles published in journals indexed in the Social Sciences Citation Index (SSCI) and Emerging Sources Citation Index (ESCI), as well as in Scimago Journal & Country Rank (SJR-Scopus). The keywords used were “dialogic feminism,” “other women,” and “women” combined (AND/OR) in the title, abstract, and article with “successful educational actions,” “dialogic gatherings,” “dialogic literary gatherings,” “dialogic model of violence/conflict prevention and resolution,” “interactive groups,” “family involvement,” “family participation,” “educative participation,” “extending the learning time” (AND/OR), “egalitarian dialogue,” “cultural intelligence,” “transformation,” “instrumental dimension,” “creation of meaning,” “solidarity,” and “equality of differences.” Figure 1 represents the PRISMA Flow diagram for the search and screening process.

#### 3.2.2 Analysis and data charting process

A systematic analysis process has been developed based on an in-depth reading of each article. Supplementary Table S1 identifies the research methodology, the context, and the profiles of the participants for each of the selected articles. Most studies use qualitative methodology with a communicative approach in an urban context. Various data collection techniques, such as daily life stories, narratives, interviews, focus groups, and observations, are used. Five studies are literature reviews, one theoretical analysis, one content analysis, and four mixed methods. Most of the studies are from Spain, although Colombia, Finland, Lithuania, Malta, Mexico, Portugal, Romania, Scotland, the UK, and the USA are also represented. Only some studies focus exclusively on samples of women without academic qualifications, but this profile of women is prioritized along with others.

#### 3.2.3 Empirical fieldwork

Life stories (LS) and focus groups (FG) have been carried out with women participants (Pa1, Pa2, Pa3, Pa4, Pa5, Pa6, Pa7, Pa8, Pa9, Pa10, Pa11, Pa12, Pa13) in associations that are part of FACEPA (adult education (AE) participants) and mothers who contribute to SEAs in the Elementary School that is a Learning Community (elementary school (ES) mothers), five interviews (I) with educators (E1, E2, E3, E4, and E5) with different profiles (AE—educator, ES teachers, headteachers, and principal). Table 1 details the profile of the participants, the data collection techniques, and the SEAs in which they are involved. The data collection techniques have been carried out from a communicative approach in which the hierarchy between researcher and researched disappears, developing a dialogue in which the researcher provides existing scientific evidence on the issues being addressed and the researched person provides experiential knowledge; jointly agreeing on the interpretations that are considered valid (Gómez et al., 2011). In this case, it should be noted that both educators and women participants share dialogic training spaces in which they read and discuss the main scientific contributions and evidence on SEAs and educational and social issues that they decide on. This fact makes the dialogue between researchers and participants much more equal.

**Table 1:** Participants’ profile.

Code	Profile	Gender	Age	Birth Country	ISCED*	Tech.	SEA
Pa1	AE participant	Female	80–85	Spain	L1	LS	Dialogic spaces Dialogic Gatherings
Pa2	AE participant	Female	75–80	Spain	L1	LS	Dialogic spaces Dialogic Gatherings
Pa3	AE participant	Female	70–75	Spain	L1	LS	Dialogic spaces Dialogic Gatherings Collaborator in dialogic literacy with immigrants.
Pa4	AE participant	Female	70–74	Spain	L1	FG	Dialogic spaces
Pa5	AE participant	Female	75–80	France	L2	FG	Dialogic spaces
Pa6	AE participant	Female	70–74	Spain	L1	FG	Dialogic spaces
Pa7	ES mother	Female	45–49	Spain	L2	FG	Interactive groups
Pa8	ES mother	Female	35–39	Spain	L2	FG /LS	Interactive groups Dialogic Literary Gatherings
Pa9	ES mother	Female	45–49	Spain	L2	FG	Interactive groups Dialogic Literary Gatherings
Pa10	ES mother	Female	40–44	Bolivia	L2	FG / LS	Interactive groups Dialogic Literary Gatherings
Pa11	ES mother	Female	45–49	Spain	L3	FG	Interactive groups
Pa12	ES mother	Female	35–39	Morocco	L1	LS	Interactive groups Dialogic Literary Gatherings
Pa13	ES mother	Female	40–44	Morocco	L1	LS	Interactive groups Dialogic Literary Gatherings
E1	AE educator	Female	45–49	Spain	L6	I	Dialogic spaces
E2	AE educator	Female	40–44	Chile	L5	I	Dialogic spaces
E3	ES teacher	Female	45–49	Spain	L5	I	Interactive groups Dialogic Literary Gatherings
E4	ES headteacher	Female	45–49	Spain	L5	I	Interactive groups Dialogic Literary Gatherings
E5	ES principal	Female	45–49	Spain	L5	I	Interactive groups Dialogic Literary Gatherings

*ISCED, International Standard Classification of Education (UNESCO Institute for Statistics, 2012). NBS, no basic studies; L0, pre-primary education; L1, primary education 1–6; L2, lower secondary education 1–4; L3, upper secondary education 1–2; L4, postsecondary non-tertiary education; L5, first stage of tertiary education 1–3/4; L6, second stage of tertiary education ½.

The selection criteria for the profiles are the following: 1. Women without higher academic qualifications who have contributed to the educational actions promoted by FACEPA and the Schools as Learning Communities; 2. Educators with a track record in the democratic education movement for adults and principals, headteachers, and teachers at the Elementary School as a Learning Community. In the case of the women participants in adult education, all of them were older than 70 years because the case study required a historical perspective. Therefore, the stories are collected from women with a long history in the democratic adult education movement. All of them were involved in adult education in their 30s, 40s, or 50s until now.

The information analysis aims to identify the action, its social impact on women, their families and communities, and its contributions to feminism. Due to the limited length of the article, Verbatim quotations from the stories, interviews, and focus groups collected as evidence are only a few examples of the contributions the women participants themselves have most supported.

## 4 Results from the systematic literature review

Supplementary Table S2 summarizes the social impact identified by each of the educational actions the “other women” have contributed, classifying them into improvements in themselves, their families, their communities, and their contributions to feminism where there is evidence of this.

### 4.1 Dialogic spaces (Roma women students’ gathering, women groups, mutual support groups)

#### 4.1.1 Impact on themselves

In the last two decades, Roma associations have been working to reverse centuries of educational segregation of the Roma people in Spain, very few of whom have access to higher education, and even fewer if they are women (Sordé et al., 2014). Among these, the action for which evidence of its social impact has been collected is the “Roma women student gatherings” (known as “Trobades” in Catalan), organized by the Roma Association of Women Drom Kotar Mestipen. These gatherings are characterized by their dialogical perspective. Roma women and the association’s workers organize the annual meeting, which brings together more than 300 participants. Roma girls and women of all ages share their needs and interests in education and learn about the experiences of Roma women who have accessed university. After attending the meeting, many participants consider new educational projects that have led many girls to continue studying until they reach university or women without basic academic qualifications to train and acquire skills that prepare them for the labor market and university (Aiello et al., 2019).

#### 4.1.2 Impact on their families

These meetings of Roma girls and women have also had an impact on generating positive expectations for their children’s education (Aiello et al., 2019), for example, by promoting solidarity networks among them that have allowed them to organize themselves so that all children could have access to electronic devices with the internet and support with online school tasks during the COVID-19 pandemic lockdown (Aiello et al., 2022).

#### 4.1.3 Impact on their communities

##### 4.1.3.1 Promoting solidarity

The solidarity interactions at the first Roma women students’ gathering held online during the lockdown contributed to transforming the virtual space into the organization of mutual aid to better cope with the uncertainties generated by the pandemic (Aiello et al., 2022). These meetings also strengthen the associative life of Roma women, generate new mobilizations, and often make women previously in the shadows become community leaders (Aiello et al., 2019). Other informal meetings between Roma women from Spain and Roma migrant women from Romania in situations of greater vulnerability have contributed to building alliances of solidarity, helping the migrant women access basic social facilities (Sordé et al., 2014), as well as contributing to overcoming stereotypes between sub-groups within the Roma population (Sordé Martí et al., 2012).

Dialogic spaces between women in the prevention of gender violence have also been studied, which have contributed to overcoming the stereotype of female rivalry as a “natural” characteristic of women, strengthening solidarity between women as one of the fundamental elements for the prevention of gender violence (Pulido et al., 2014). Interactions of solidarity have also been identified in a self-help group set up by a Muslim women’s association in a Scottish town, which is helping those who were even more isolated to enter the labor market (Ruiz-Eugenio, 2016).

On the other hand, Puigvert and Elboj (2004), from her involvement with the “other women” in an adult school where dialogic feminism is promoted, analyses how this school has been the place where “other women” have created dialogic spaces where they have defined and pursued their educational interests, while at the same time promoting a solid social network in which they express their personal, educational, and social concerns.

### 4.2 Dialogic Gatherings (literature, science, mathematics)

#### 4.2.1 Impact on themselves

Several studies showed that the involvement of women without academic qualifications, some of whom are immigrants or belong to cultural minorities, in Dialogic Literary Gatherings (DLG) improves their self-confidence in their own academic, cultural, and communicative abilities (De Botton et al., 2014; Duque, 2015; Garcia Yeste et al., 2017b; Flecha, 2015) and the language skills (De Botton et al., 2014), as well as their involvement at the Dialogic Mathematics Gatherings improves their mathematics skills (Díez-Palomar, 2020), and the Dialogic Scientific Gatherings overcome their difficulties to access scientific knowledge, fostering analytical and critical thinking based on scientific evidence, using scientific knowledge in decision-making on their health-related habits (Buslón et al., 2020), and improving their raising awareness about sciences behind the social impact of education and gender research (Ruiz-Eugenio et al., 2023a).

#### 4.2.2 Impact on their families

There is also evidence that the involvement of Muslim immigrant mothers without basic academic qualifications in Dialogic Literary Gatherings is increasing learning and reading interactions at home, increasing their children’s reading and learning motivation (De Botton et al., 2014); as well as the Dialogic Scientific Gatherings contribute to women participants using the scientific knowledge in decision-making on their and their family’s health-related habits (Buslón et al., 2020).

#### 4.2.3 Impact on their communities

Other studies have demonstrated that DLG is an educational action that positively impacts the community by helping to overcome racist prejudices against Muslim immigrant women, creating a supportive environment, and strengthening social networks (Garcia Yeste et al., 2017a; Ruiz-Eugenio et al., 2021). Additionally, through Dialogical Scientific Gatherings, the participating women are promoting solidarity and active citizenship in response to the challenges of today’s society (Buslón et al., 2020).

### 4.3 Dialogic model of conflict prevention and resolution

#### 4.3.1 Impact on themselves

Two studies have focused on the impact of the involvement of “other women,” primarily mothers, in the dialogic model of conflict prevention and resolution in their children’s schools (Serradell et al., 2020; Oliver et al., 2009). This model is based on including the whole community, especially students and their families, in deciding on the rules of coexistence, jointly identifying the causes and origins of conflicts and their solutions, and focusing on prevention. Mothers, even older sisters, aunts, and grandmothers, contribute to the assemblies and commissions created for the prevention of conflicts and gender violence, being very active in the identification of situations and in the joint elaboration with the teaching staff and other professionals of the actions to be carried out (Oliver et al., 2009; Serradell et al., 2020).

#### 4.3.2 Impact on their families

The dynamic of discussing the rules that everyone has agreed upon at school is transferred to their homes, impacting their children’s behavior and reinforcing the norm of coexistence agreed upon by the community (Serradell et al., 2020).

#### 4.3.3 Impact on their community

In this model, the “other women” are not only in the decision-making spaces but also in the learning spaces. Immigrant mothers from Morocco contribute to the interactive groups, a way of organizing the classroom in small heterogeneous groups where an adult dynamizes learning interactions. The fact that these mothers are inside the classrooms as volunteers in the interactive groups has had an impact on improving the behavior not only of their children but also of all the children (Serradell et al., 2020).

### 4.4 Family education and educative participation

#### 4.4.1 Impact on themselves

Several studies have provided evidence that immigrant women, who do not know the language of the host country or have no basic education, participating in family education programs in their children’s schools have had an impact on their academic and linguistic skills, improving their self-confidence to help their children with homework (Girbés-Peco et al., 2019; Flecha, 2012). Some of these family education actions are the aforementioned DLGs, as well as literacy courses and reading and writing programs in Spain, Malta, the UK, and Mexico (Flecha, 2012; Ocampo-Castillo et al., 2023), and the courses for obtaining the Compulsory Education for Adults Graduate (Garcia Yeste et al., 2018). Participation in family education programs has also led many of these women to participate in other spaces as mixed commissions where families, teachers, and students decide on crucial issues related to educational and school improvement, and the interactive groups in the classroom of their children (Garcia-Carrion et al., 2018), In interactive groups, the “other women” contribute to increase the learning interactions among the students (Garcia-Carrion et al., 2018). These women report that their involvement in these spaces has had benefits such as increasing autonomy (Girbés-Peco et al., 2019), self-esteem improvement (Garcia Yeste et al., 2018; Girbés-Peco et al., 2019; Ocampo-Castillo et al., 2023), and learning motivation and expectations for themselves (Garcia Yeste et al., 2018, 2019).

#### 4.4.2 Impact on their families

There is also abundant evidence of the benefits for their families, especially their children, of the participation of these women in family education activities (literacy, health education, among others) and their contribution to educative participation spaces such as mixed committees and interactive groups. The benefits reported by the teachers, the women themselves, and the students were improving their children motivation for learning and expectations (Flecha, 2012; Flecha and Soler, 2013; Garcia Yeste et al., 2012; Renta Davids et al., 2018; Rodríguez-Oramas et al., 2022), school absenteeism reduction (Flecha, 2012; Flecha and Soler, 2013; Rodríguez-Oramas et al., 2022; Soler et al., 2019), the elimination of dropout in primary education (Garcia-Carrion et al., 2018), improving their children reading and mathematics outcomes (Flecha and Soler, 2013; Renta Davids et al., 2018; Rodríguez-Oramas et al., 2022), transforming classroom interactions (Flecha and Soler, 2013), improving children’s behavior at school (Renta Davids et al., 2018; Rodríguez-Oramas et al., 2022), increasing learning interactions at home (Renta Davids et al., 2018; Flecha, 2012; Garcia Yeste et al., 2018), increasing children’s participation in extended learning time activities (Renta Davids et al., 2018), and improving their children’s health and health-related family habits (Flecha et al., 2011; Ruiz-Eugenio, 2016).

#### 4.4.3 Impact on their community

It has been identified that the dialogic approach in educative participation contributes to overcoming the folkloric view of the involvement of cultural minority families that often stems from social and cultural stereotypes in the community (Díez et al., 2011; Flecha and Soler, 2013). Teachers provide opportunities for these families and other community members to volunteer in learning environments with their children, e.g., interactive groups and decision-making spaces such as assemblies and mixed committees (Díez et al., 2011; Flecha and Soler, 2013). The “other women” are the majority of those in these families who are involved in these learning environments and decision-making spaces, contributing to overcoming in the whole community the stereotypical beliefs about the skills of women without academic education, immigrant women, or those from cultural minorities (Garcia Yeste et al., 2012; Christou and Puigvert, 2011). The participation of these women in the school contributes to their reinforcement of social networks, creating an environment of supportive relationships in the school and the community (Garcia Yeste et al., 2018, 2019; Girbés-Peco et al., 2019; Ocampo-Castillo et al., 2023), and contributes to the building of trust between families and school’s staff (Khalfaoui et al., 2020). The “other women” involvement in learning and decision-making spaces positively impacts the school and community climate (Flecha and Soler, 2013; Rodríguez-Oramas et al., 2022; Christou and Puigvert, 2011). It has also been studied how the involvement of the “other women” in deciding on commissions aimed at improving the health of their children’s schools has had an impact on the promotion of health literacy activities that have been successful in participation because they responded to the needs expressed by women (Flecha et al., 2011) as well as better prevention and early detection of drug abuse has been carried out (Flecha et al., 2011).

### 4.5 Extending the learning time

#### 4.5.1 Impact on themselves

The involvement of girls from immigrant backgrounds or cultural minorities such as the Roma in after-school learning extension spaces has contributed to reducing their school dropout rates in secondary education, increasing their access to post-compulsory and university studies (Ruiz-Eugenio, 2016; Pascual et al., 2020).

#### 4.5.2 Impact on their families

The “other women” engagement, such as mothers and grandmothers, in extended learning time at the tutorized library in their children’s school has improved children’s learning and achievement (Flecha and Soler, 2013; Rodríguez-Oramas et al., 2022).

### 4.6 Access to higher education

#### 4.6.1 Impact on themselves

It has been studied how the support networks created among Roma women who access university help them continue their studies and not drop out (Pascual et al., 2020).

#### 4.6.2 Impact on their families

The climate of support transmitted in these solidarity networks leads women to develop more supportive attitudes toward their children by expanding their educational opportunities (Pascual et al., 2020).

#### 4.6.3 Impact on their communities

These Roma women become role models for Roma girls as key agents of change in the Roma community (Pascual et al., 2020).

### 4.7 Contributions to feminism

The literature shows that dialogic spaces in adult education are where the concept of “other women” and the dialogic feminism theorized by Puigvert in 2001 with the “other women” arose, in particular with the FACEPA Women’s Group and the La Verneda-Sant Martí Adult Education School, especially in its participating women’s association Heura (Puigvert and Elboj, 2004). Building on Puigvert’s theorization of dialogic feminism and the contributions of the “other women” to feminism, other studies have focused on the contributions of these women to the prevention of gender violence, specifically on how the dialogic basis of the FACEPA Women’s Group contributes to creating knowledge from the everyday life of the “other women” in dialogue with contributions from international research, having a direct social impact on these women (Valls, 2014). The work of women educators with the “other women” has also been studied, with the former making themselves available to the latter’s needs and interests (Arrufat, 2004). Other studies have focused on including the voices of the “other women” on an equal basis with women researchers throughout the process of developing research that directly affects them (Garcia Yeste et al., 2011; Ramis et al., 2014). It has also collected life stories of women who have become literate and then participated in the DLGs, being among the participants who have been most involved in their community, claiming the rights of all women to education, being very active in demanding the inclusion of “other women” in feminism (Flecha, 2015).

Other studies have shown how the dialogic model of conflict prevention and resolution includes the “other women” along with female educators and teachers in the creation of actions for the prevention of gender violence at school (Oliver et al., 2009), contributing to improving the school and community climate (Serradell et al., 2020).

Studies on Roma feminism have shown that within the framework of Puigvert dialogic feminism, non-academic Roma women have recognized themselves as feminists (Sordé et al., 2014). The demands of dialogic Roma feminism seek not only the overcoming of gender inequalities but also respect and equality for all Roma and their culture in society and the spaces of feminist debate (Aiello et al., 2024).

Other studies on Roma feminism have shown the creation of solidarity networks between Spanish Roma women and immigrant Roma women (Sordé et al., 2014). The Spanish Roma women have supported the migrant Roma women from Romania to enter the social services system, continue their studies, and find or create their jobs by taking the initiative themselves (Sordé et al., 2014). It has also been identified that Roma women’s access to university has facilitated a dialogue between Roma and non-Roma women that has contributed to overcoming stereotypes about Roma culture, including the vision of the role of families in supporting education. This dialogue has also transmitted the value of being a Roma woman and feminist who fights for equality without renouncing her culture (Pascual et al., 2020).

## 5 Results from the fieldwork

From the interviews with educators and teachers, life stories, and focus groups with women participants, evidence of the social impact the “other women” has contributed, especially in the educational actions of Dialogic Gatherings and other dialogic learning spaces, has been identified. Specifically, evidence of the social impact of their contributions to social transformations in their neighborhoods, in the co-creation of the democratic adult education movement, in the transformation of spaces of participation that exclude non-academic women, in the co-creation of the project “Thousand and One Dialogic Gatherings Around the World,” in the prevention of gender violence and the promotion of new alternative masculinities, and in becoming feminist references for boys and girls.

### 5.1 Dialogic Gatherings and other dialogic learning spaces

#### 5.1.1 Impact on themselves

##### 5.1.1.1 Improved confidence in their abilities

Eight of the stories collected have participated in DLG, and all of them agreed that this educational action had contributed most to their self-confidence, their loss of the fear of speaking in public, and to learning to develop argumentation. Pa3 has been participating in a DLG for ten years; she started attending when she became a widow. In her story, she explains how she reads books she never imagined she could read and how reading them opened a whole new world for her. She began to believe in her abilities and to overcome the self-perception conveyed by dominant discourses in our society that people without academic qualifications cannot read and enjoy the best literary creations:

[In the DLG] I dared to read books I would never have read alone (…) But reading with my classmates opened a world for me. First, to have that confidence I did not have in myself (Pa3_AE_LS).

Pa1 has been involved in DLG for over 30 years; she also started attending adult education in 1992 when she became a widow. Then she graduated in basic education. She stresses that the atmosphere created in DLG by the egalitarian dialogue in which all contributions are valued for the arguments on which they are based and the respect for all people helps them feel more confident to intervene:

We read books like Don Quixote, One Hundred Years of Solitude, or Ulysses by James Joyce. At the tertulia, people get used to speaking in public because of our reading system at home, and then everyone can comment on what they think in dialogue. You contrast your opinion with other people, and apart from that, you learn to speak in public without shame; it is very important, as it gives you much security that when you speak, people will not laugh at you (Pa1_AE_LS).

#### 5.1.2 Impact on their families

##### 5.1.2.1 Contributing to the prevention of gender violence and the promotion of new alternative masculinities

Pa1 has also been involved for many years in the women’s group of FACEPA. This group meets monthly to discuss topics of their choosing related to women’s equal rights. Discussions usually start after a short presentation of the topic. These presentations are elaborated by educators and women participants and are based on scientific evidence of social impact. For example, some of the topics worked on are the educational actions that contribute to the prevention of gender violence and the role of new alternative masculinities in overcoming it. In this framework, they have worked on how to promote new alternative masculinities from early childhood, giving much security to those boys who relate without using violence and always defending their peers. In her story, Pa1 explained how, before she knew what an alternative masculinity to dominant masculinity was, she was already promoting this model at home with her children. Specifically, she explained how she has always tried to get both her daughter and son to assume equality between men and women, to take equal responsibility for household chores, and to take a stand against violence:

We call it new alternative masculinities. I have a son and a daughter, and I have cared that they have always been equal. My son has always been for women’s rights and against violence. When I go to my son’s house and see that he is ironing, I do not say, “Let me iron for you.” We mothers have influenced all these things. Luckily, there are new masculinities because not all men are sexist (Pa1_AE_LS).

Some are role models for their children, nieces, nephews, or grandchildren in promoting and choosing more egalitarian relationships. In the accounts collected from both Elementary School as a Learning Community mothers and teachers (Pa8, Pa11, E3, E4, E5) and women participants in adult education (Pa1, Pa2), having participated in dialogue spaces about evidence of social impact for violence prevention, whether in Dialogic Gatherings or other dialogic spaces, has helped them to have conversations with their children or grandchildren about the relationships they have with their peers. One of the themes that the participants pointed out that contributed to these conversations is the value of true friendship, which will always be free of violence, coercion, or abuse. Another theme that they said has helped them in conversations with their family members is the identification of the dominant coercive discourse through the messages that come to us both from everyday relationships and from social networks, media, and films that show desire toward people who use violence of any kind.

#### 5.1.3 Impact on their communities and beyond

The social impact in their communities of the actions to which the “other women” have contributed ranges from the social transformation of their neighborhoods to the creation of a movement of democratic education and Dialogic Gatherings around the world.

##### 5.1.3.1 From a shantytown to the best library in the world

The “other women” and, especially, the participants in the DLGs have been the majority of the people involved in the demands for improving their neighborhoods. Pa1 explains how the adult school in which she has been engaged for more than 30 years has driven social transformation in her neighborhood. Achieving better living conditions in the neighborhood has always been one of the priorities of this adult school. In her story, she explains how the school creates spaces not only for learning but also for participation in deciding how to deal with situations of inequality in the neighborhood; spaces in which both people who are learning to read and write or learning the language and educators participate:

Through coming to the school, contacting so many women, people who had not had the opportunity to study before but who were very smart, by coming together here at the school, in the gatherings, you are encouraged to share… it is very important for society to communicate and for women to communicate with each other; it is a neighbourhood where we have achieved a lot through coming together and organising ourselves (Pa1_AE_LS).

In the 1970s, this neighborhood still had a large area of shantytowns. The mobilization of the participants in this school, mainly the “other women,” has made possible improvements such as the disappearance of the shanties, the creation of housing, the arrival of the metro and many bus lines, the recovery of the abandoned farmhouses for social, educational, and cultural services, the creation of a large park and a 2.5-km-long tree-lined boulevard where a motorway was to be built as well as the one that has been named the best new library in the world in 2023 by the International Federation of Library Associations and Institutions. Pa1 referred to this: *We had a shantytown, and now we have the best library in the World* (Pa1_AE_LS).

##### 5.1.3.2 Co-creation of the democratic adult education movement

The “other women” have promoted a model of democratic adult education, where the participants, those who do not have a university degree, are part of all decision-making spaces, being the protagonists of their training processes. This movement, which has spread internationally, is co-created by participants in adult education, educators, and researchers; the latter two groups put their knowledge and work at the service of the participants to overcome the social and educational inequalities that they suffer because they have not been able to access education and obtain academic qualifications.

Within this movement’s framework, adult education participants’ associations are created. Some are mixed associations of men and women; others are associations exclusively for women, which aim to address not only women’s right to education but other specific issues that affect them because they suffer from gender discrimination. Educator 1 has been involved in the democratic adult education movement since the 1990s. She explains how the “other women” were the majority of those who drove the democratic adult education movement; how these women’s associations are created in many neighborhoods and villages, being not only spaces in which they will decide on educational projects and activities, but also becoming spaces for the creation of networks of solidarity and friendship among the women themselves:

They created the movement. They had no academic qualifications; they went to entities, associations, and spaces to learn and increase their academic and educational level. This was a very important change for them in their lives because it is a space where they go to learn, where they dialogue, where they make friends, and where many solidarity networks are generated (E1_AE_I).

In the focus group, Pa5 and Pa6 participants from one of these associations explain how in their neighborhood, like many others in the city of Barcelona, in the 1960s, many people had arrived from other parts of Spain, mainly from rural areas, to work in the city’s industry. These neighborhoods on the outskirts of Barcelona were created to accommodate this population. Many women needed to be literate. This was one of their association’s main objectives. Later came other such as learning ICTs. The neighborhood’s women who could read and write taught the others; later, this also happened with ICTs; this was explained in the focus group by two of the participants:

Pa6: It is a neighbourhood that had emigrants from all over Spain. This association was created because we saw the need to bring culture to many women who, at that time, had not been able to go to school when they were children, so they did not know how to write or read. This was the first objective of the association (…) (P6_AE_FG).

Pa5: I learned about computers, and then, with the little I knew about computers, I wanted to pass them on to women because I thought they were important (P5_AE_FG).

Spaces are created for these women from different neighborhoods and towns to meet. The creation of FACEPA, the Federation of Cultural and Educational Associations of Adults, in Catalonia in 1996 was critical to the organization of this movement. Educator 1 explains how they started to create these meetings and helped each other to create their associations in different neighborhoods and towns as a way of claiming their rights in education:

They start to unite and help each other from different neighbourhoods to gain legitimacy and legality. They create women’s associations (…). They get together to organise themselves and start grouping. As they do this, they claim their voice in important debate spaces, such as in education (E1_AE_I).

That same solidarity created among the “other women” who had not had the opportunity to access education when they were children is what they have given to immigrant women who have arrived decades later in their cities. Pa3 has been a volunteer in the literacy class for several years, where most participants are now immigrant women and men who arrived in Barcelona not only without knowing the language of the host country but also without being literate in their languages. Pa3 explains how she feels that with everything she has received in this school, she now must give to those who need it most: *the school offers you this space to give as well as to receive, so you get involved; it’s not just about receiving; if they have given me, then whatever I have, if I can, I also give it* (Pa3_AE_LS).

##### 5.1.3.3 Transforming spaces for participation that exclude non-academic women

FACEPA has coordinated many European projects in which spaces for participation are created, driven by women who, because they do not have academic qualifications, had been left out of the spaces for public debate and decision-making on the educational projects aimed at them. It has also participated as a partner in European projects run by other organizations. The democratic education model has been extended in Europe through these projects. Educator 2 explained in her interview that it is still difficult today for the technical staff of these associations to understand that a project aimed at non-academic women can only be promoted with them having been involved in the whole decision-making process, from its design, development, and evaluation. The educator explains an actual situation to illustrate this. In one of the projects they are part of as a partner entity on citizen participation, people who did not know English were not allowed to participate in the meetings. Therefore, the people who were supposed to be the project target were excluded from participation. The women of FACEPA explained to the coordinating body how the associations of their federation do it. The previous spaces are created where the participants and the educators prepare the content, and there is always a support person who translates what they want to present. They are organized in small groups in the meetings in which the person who knows English translates both what other people are presenting and what they want to present to the audience. This input from the “other women” was crucial for the project’s coordinating organization, changing how they organize the meetings. They now include the final beneficiaries of the project in all meetings, as they do in FACEPA. Educator 2, after explaining this example, focuses on how it has been the “other women” who have promoted this change in the technical staff of organizations in other European countries.

It also generates changes in the technical teams working in adult education in Europe, now including the participants in all decision-making processes (E2_AE_I).

##### 5.1.3.4 Co-creating the thousand and one Dialogic Gatherings around the world project

The “other women” were vital in co-creating the “Thousand and One Dialogic Gatherings Around the World” project. The first Dialogic Gathering was created in the Adult Education School of La Verneda-Sant Martí, Barcelona, in the 1979–1980 academic year. After more than 17 years of participating, Ramón Flecha published the book Sharing Words, in which he theorizes the dialogic learning on which the DG is based (Flecha, 1997). This book was soon translated into English (Ruiz-Eugenio et al., 2023b; Padrós-Cuxart et al., 2024), among other languages such as Chinese. At the same time, in co-creation with the participants, the majority of whom were non-academic women, FACEPA promoted the “Thousand and One Dialogic Gatherings Around the World” project to disseminate and transfer this educational action. Almost 50 years after the creation of the first DG, there are more than 15,000 DG transferred from adult education to preschool, primary, secondary, and special education, as well as to other social, cultural, and educational entities such as residential centers for minors, prisons, hospitals, and primary health care centers, among others (Ruiz-Eugenio et al., 2023b; Padrós-Cuxart et al., 2024). Educator 1 and participants Pa1 and Pa2 explain that in the early years, the women participating in the first DLG went around towns and cities to explain how the DLGs were carried out and the impact they had had on their lives:

They created the Dialogic Gathering movement, which comprised both men and women, but women were significant because they were the majority in the gatherings. Therefore, they were the ones who went. They took a train, and they could go to Castilla La Mancha, Galicia, and Andalusia to explain what the Dialogic Gatherings were; they did it accompanied by a female educator (E1_AE_I).

##### 5.1.3.5 Women participants in the DLGs are feminist role models for children

Parallel to the extension of the DLGs by schools and adult education associations, this educational action was transferred to preschool, primary, and secondary education, which is one of the successful educational actions of Schools as Learning Communities. In this transfer, in addition to the researchers of the research center that created the Schools as Learning Communities project (Giner, 2018; Soler Gallart, 2017), the women participants, mainly from the first DLG, have visited schools to explain to them how it works. The “other women” have contributed to the fact that thousands of children worldwide have learned and enjoyed reading and discussing the best literary creations, which, according to the reproduction theories of the sociology of education, were reserved only for the elites. Educator 2 explained this in her story:

The participating women are very clear about this. Fortunately, they helped us maintain the criteria. They are clear that not just any reading has the same impact as one of humanity’s greatest literary creations. They always focus on meeting these criteria. When the women participants present the Dialogic Literary Gatherings, they first ensure that. It has an impact that has allowed the children of the world to have a much higher quality education with the DLG (E2_AE_I).

DLGs take place in preschool, accelerating literacy skills. The women who contributed to creating DLGs are role models for many children. Some teachers who are carrying out DLGs in early childhood education have worked on feminist role models in their classes by also including anonymous women such as their mothers, grandmothers, and neighbors. In one of these schools, a teacher in the 4-year-old preschool class promoted that, to celebrate International Women’s Day on March 8, whoever wanted to come dressed up as a woman who had done something significant could do so. One of the girls dressed up as one of the women participants of the first DLG (Pa2 of this study). Pa2, after being taught literacy, started to participate in the DLG and be one of the most involved in transferring this educational action. The girl wore reading glasses and a jumper of the same color as this woman in one of the pictures of her on the internet; she also carried a book in her hands. This girl did not know this woman in person, but the way the teacher explained to the children what DLGs are, why and how they were created, and how to enjoy them themselves made this girl have no doubts about which woman to choose. A few days later, the teacher facilitated a video conference with Pa2. The mothers of some of the children also wanted to participate. Educator 2, who, together with the teacher, managed the video call, explained the excitement of both the children and the woman participant:

They made a video call so the children could meet her. It meant the children were talking to a woman they admired. It was very, very exciting because she is a role model. What touched Pa2 the most was that she was considered a point of reference not only because of the gatherings but also because she was a feminist reference. It was her commitment to society, equality, and making the voice of the “other women” heard for so many years. She was not only a point of reference for doing gatherings but also a point of reference as a feminist woman, which was very nice (E2_AE_I).

## 6 Discussion

The systematic literature review provides evidence of the emergence of dialogic feminism in the dialogic spaces in adult education, especially those promoted by FACEPA and the Adult School of La Verneda-Sant Martí, in which Lidia Puigvert participates together with the “other women” (Puigvert, 2001a; Puigvert and Elboj, 2004). The review has identified the contributions that Puigvert’s dialogic feminism makes to sociological theory and other social and humanities sciences (Puigvert and Flecha, 2010; Beck-Gernsheim et al., 2003; Di Fanti et al., 2022) as well as research on the inclusion of the “other women” in the spaces of public debate on feminism and education (Puigvert and Elboj, 2004; Arrufat, 2004; Garcia Yeste et al., 2011; Ramis et al., 2014; Valls, 2014; Ruiz-Eugenio et al., 2023c; Christou and Puigvert, 2011). Most of the articles reviewed focus on the social impact of educational actions to which the “other women” have contributed, providing evidence of improvements in their lives, families’ lives, and communities. Among these, studies that have analyzed different dialogic spaces, such as Roma Women Students’ Gatherings (De Botton et al., 2014; Duque, 2015; Garcia Yeste et al., 2017b; Flecha, 2015; Ruiz-Eugenio et al., 2023b), and the Women Groups in adult education (Valls, 2014), as well as the Roma feminism based on dialogic feminism (Sordé Martí et al., 2012; Sordé et al., 2014; Aiello et al., 2024).

The educational actions to which the “other women” have contributed and for which the most evidence of their social impact has been collected in the literature review are family education—(literacy, writing and reading programs, basic education, and health education) and educative participation (family involvement in interactive groups in the children’s classrooms and decision-making spaces such as mixed committees and assemblies in their children’s schools). Most of these studies have analyzed the social impact of family education and educative participation actions (Flecha, 2012; Flecha and Soler, 2013; Garcia Yeste et al., 2012, 2018, 2019; Renta Davids et al., 2018; Rodríguez-Oramas et al., 2022; Díez et al., 2011; Melgar et al., 2011; Gómez-González et al., 2024; Girbés-Peco et al., 2020; Girbés-Peco et al., 2019; Ocampo-Castillo et al., 2023; Garcia-Carrion et al., 2018; Khalfaoui et al., 2020; Flecha et al., 2011; Ruiz-Eugenio, 2016).

The studies focused on Dialogic Gatherings, although some of them are part of family education actions in which the “other women” are involved in their children’s schools, have been analyzed in a specific category for this action as it is one of those for which the most evidence of its social impact has been collected and in which “other women” participate in other places than their children’s schools, such as adult education schools and associations. Among these, the Dialogic Literary Gatherings are the action for which the most evidence of its impact has been collected (De Botton et al., 2014; Duque, 2015; Garcia Yeste et al., 2017b; Flecha, 2015; Ruiz-Eugenio et al., 2023b). Studies have also been carried out on the contributions of the “other women” in the Dialogic Mathematic Gatherings (Díez-Palomar, 2020) and in the Dialogic Scientific Gatherings (Buslón et al., 2020; Ruiz-Eugenio et al., 2023a). Two articles have focused on the contributions of the “other women” to the Dialogic Model of Conflict Prevention and Resolution (Oliver et al., 2009; Serradell et al., 2020), and two others on actions related to the extension of learning time, such as the tutorized library in which mothers and grandmothers have contributed to support children in reading or with school homework (Flecha and Soler, 2013; Rodríguez-Oramas et al., 2022; Ruiz-Eugenio, 2016), as well as the Roma “other women” have been crucial in supporting their daughters to continue their education beyond compulsory schooling and to graduate from university (Pascual et al., 2020).

Regarding the limitations of the systematic literature review, it is important to note that the studies are primarily conducted in Spain, although other countries such as Colombia, Finland, Lithuania, Malta, Mexico, Portugal, Romania, Scotland, the UK, and the USA are also represented. This bias is due to the fact that studies on the social impact of educational actions to which the ‘other women’ have contributed within the framework of Schools as Learning Communities and democratic adult education have been mainly developed in Spain. This is because Spain is the country of origin for the conceptualization of dialogic feminism and dialogic learning, which underpin these actions and the movement of the ‘other women.’ Over the past two decades, these educational actions have spread worldwide, particularly in Latin American countries. However, there is a limitation in the number of studies published in international databases from these regions. More research is needed to enrich this systematic review by broadening the scope.

In terms of the fieldwork conducted in the case study, the importance of including the historical perspective of the contributions of the “other women” has led to the collection of further evidence from the life stories and the focus group with the women participants and the interviews with the two adult education educators who have been involved for decades. Some of them experienced first-hand the origin of dialogic feminism and the democratic adult education movement, also analyzed in both the systematic literature review (Puigvert, 2001a; Puigvert and Flecha, 2010; Puigvert and Elboj, 2004) and the contextualization of the case study (Beck-Gernsheim et al., 2003). In the narratives with these women participants and the interviews with the educators, there is evidence of how the “other women” contributed to the social transformations in their neighborhoods from their adult education associations. They were also the majority in creating a democratic adult education movement and the “Thousand and One Dialogic Gatherings Around the World” project. They were also involved in the transfer of the DLGs to primary and secondary schools. The DLGs are also the educational action for which the most evidence has been collected as the actions that have generated the most transformation in themselves, their families, and communities.

In the stories of both the AE participants and the ES mothers, as well as the interviews with AE educators and ES teachers, it has become evident how the participation of the “other women” in different dialogic spaces, such as the gatherings or the women’s groups, has contributed in which they have read and discussed the scientific evidence of social impact on the prevention of gender violence and the new alternative masculinities. The “other women” have transferred these dialogues to their families, promoting reflection among children, nephews, and grandchildren on the importance of identifying as brave and attractive people who always stand against violence and defend the victims. These women are making a fundamental contribution in their families and communities to one of the most worrying challenges today: overcoming violence and gender violence.

## Data Availability

The datasets are not directly available online to ensure the necessary level of confidentiality and the legitimate utilisation of the data. Researchers interested in accessing any of the datasets are kindly requested to make a formal request by sending an email to Laura Ruiz-Eugenio. This request should be accompanied by the following documents: a formal letter containing the researcher’s contact information, institutional affiliation, current position, the purpose of the research, details regarding the intended use of the data, and, if applicable, information about funding sources; an official letter from the researcher’s affiliated university or research institution confirming their association; and a confidentiality agreement, duly signed by the researcher, indicating their commitment to maintaining the confidentiality of the data.
