# Supplementary material for: A systematic literature review and case study on the social impact of the other women’s contributions to education and dialogic feminism
Source: Front Sociol. 2024 Dec 9;9:1477983. doi: 10.3389/fsoc.2024.1477983 (PMC11664246; doi:10.3389/fsoc.2024.1477983)
Supplement: Supplementary file 1 [file Table_1.docx]

Supplementary Material

# Supplementary Table 1

***Characteristics of the reviewed studies***

| **N** | **Reference** | **Method** | **Context and profiles** |
| --- | --- | --- | --- |
| 1 | (Aiello-Cabrera et al., 2024) | Semi-structures interviews, n=23 | Spain, Roma women, 18-58 years old, involved in grassroots Roma civic or political organisations. |
| 2 | (Aiello et al., 2022) | Content analysis of the recording of the whole event (2 hours 15 minutes), n=25 | Spain, 19th edition of the Romana Women Students' Gathering organised by the Drom Kotar Mestipen association, held online in July 2020. Roma girls and women 9-67 years old |
| 3 | (Aiello et al., 2019) | Case study | Spain, Roma Association of Women Drom Kotar Mestipen |
| 4 | (Arrufat, 2004) | Theoretical analysis from the pedagogy of autonomy (Freire) and dialogic Feminism (Puigvert) | Spain, grassroots women's associations |
| 5 | (Buslón et al., 2020) | Communicative focus group, n=4 | Spain, urban adult school. Women without university degrees |
| 6 | (Christou and Puigvert, 2011) | Qualitative case studies | Finland, Lithuania, Malta, Spain, and the UK have urban primary schools in low SES areas with immigrant families and ethnic minorities. |
| 7 | (Corrêa di Fanti et al., 2022) | Theoretical analysis from dialogic Feminism (Puigvert) and Bakhtin |  |
| 8 | (de Botton et al., 2014) | Qualitative case study, n=20 | Spain, an urban elementary school in a low SES neighbourhood. Moroccan immigrant mothers |
| 9 | (Díez et al., 2011) | 5 Qualitative case studies, n=160 | Finland, Lithuania, Malta, Spain, and the UK have urban primary schools in low SES areas with immigrant families and ethnic minorities. |
| 10 | (Díez-Palomar, 2020) | Qualitative case study, n=8 | Spain, urban adult school. Women without university degrees. |
| 11 | (Duque, 2015) | Narratives n=5 | Spain. Women without university degrees. |
| 12 | (Flecha et al., 2011) | Qualitative case studies  Open-ended interviews with community project professionals = 13 x 2 schools  Communicative daily life stories = 13 x 2 schools  Communicative focus group with professionals working in the school = 1 x 2 schools  Communicative observations in family involvement activities = 5 x 2 schools | Spain, two urban primary schools in low SES neighbourhoods (migrant and Roma families, primarily mothers) |
| 13 | (Flecha, 2012) | Qualitative case study, n=11 | Finland, Lithuania, Malta, Spain, UK. Urban primary schools in low SES areas with immigrant families and ethnic minorities. Migrant mothers and girls with ethnic minority backgrounds. |
| 14 | (Flecha, 2015) | Narrative n=1 | Spain, urban adult school, non-academic woman. |
| 15 | (Flecha and Soler, 2013) | Longitudinal mix-method case study, n= 266 | Spain, an elementary school in a low-SES urban area. Most of them are Roma mothers and children. |
| 16 | (Garcia-Carrion et al., 2018) | Qualitative case study, n=18 | Spain, a primary and secondary school with families from cultural minorities in low-SES urban area. 12 students and relatives. |
| 17 | (Garcia Yeste et al., 2011) | Literature review |  |
| 18 | (Garcia Yeste et al., 2018) | Communicative case study, n= 8 | Spain, an elementary school in a low SES urban area. 6 mothers and 2 student girls. |
| 19 | (Garcia Yeste et al., 2012) | Qualitative case study, n= 16 | Spain, two elementary schools in low SES urban areas. Primarily mothers with no academic qualifications and from minority cultural backgrounds. |
| 20 | (Garcia Yeste et al., 2019) | Qualitative study, n=144 | Spain, 8 elementary schools with families from cultural minorities in low SES urban areas. 80 relatives, most of them mothers. |
| 21 | (Garcia Yeste, 2014) | Communicative daily life stories, n=5 | Spain, 3 elementary urban schools from low SES areas. Non-academic mothers from migrant or ethnic minority backgrounds. |
| 22 | (Garcia Yeste et al., 2017b) | Communicative life stories and observations, n=20 | Spain, adult school. Women without education degrees. |
| 23 | (Gómez-González et al., 2024) | Mix-method study, n= 209 | Spain, 8 primary urban schools in low SES areas with high rates of Roma and Moroccan families. |
| 24 | (Girbés-Peco et al., 2019) | Qualitative study, n= 108 | Spain, 8 primary urban schools in low SES areas with high rates of Roma and Moroccan families. 33 Roma and 18 Moroccan mothers. |
| 25 | (Girbés-Peco et al., 2020) | Qualitative case study  Communicative observations | Spain, primary urban school in low SES area with high rates of Moroccan families. |
| 26 | (Khalfaoui et al., 2020) | Qualitative case study, n= 12 | Spain, an elementary urban school in a low-SES neighbourhood, 6 family members, 1 Roma mother and 3 migrant mothers. |
| 27 | (Melgar et al., 2011) | Longitudinal qualitative case studies | Finland, Lithuania, Malta, Spain, UK. Urban primary schools in low-SES areas with immigrant families and ethnic minorities. |
| 28 | (Munté Pascual et al., 2020) | Communicative daily life stories, n=4 | US, Spain. Roma women from low SES families. |
| 29 | (Ocampo-Castillo et al., 2023) | Communicative daily life stories; semi-structured interviews; discussion group, n=6 | Mexico, old grandmother in literacy process. |
| 30 | (Oliver et al., 2009) | Qualitative study, n=53 | Spain, 6 urban primary schools, 28 students and their relatives, most girls and mothers. |
| 31 | (Puigvert and Elboj, 2004) | Qualitative study | Spain, women in adult education associations. |
| 32 | (Pulido et al., 2014) | Qualitative study, n=54 | Spain, 5 urban high schools. Girls 14-18 years old. |
| 33 | (Ramis et al., 2014) | Communicative daily life stories | Spain, non-academic women |
| 34 | (Renta Davids et al., 2018) | Communicative case studies, n=261 | Spain, 8 elementary schools in low SES areas, 164 students' relatives, 79% women. |
| 35 | (Rodríguez-Oramas et al., 2022) | Mix method case study, quantitative n=400 (students); qualitative n=19 (family of students) | Mexico, an elementary school in a highly underprivileged urban area. 5 mothers and 1 grandmother. |
| 36 | (Ruiz-Eugenio, 2016) | Literature review | Scotland-UK  Non-academic migrant women |
| 37 | (Ruiz-Eugenio et al., 2023a) | Qualitative case study, n=12 | Spain, an urban elementary school in a low-SES area. 10 mothers. |
| 38 | (Ruiz-Eugenio et al., 2023c) | Literature review | Spain  Non-academic women |
| 39 | (Ruiz-Eugenio et al., 2023b) | Systematic literature review | Mexico, Spain, UK  Non-academic women |
| 40 | (Ruiz-Eugenio et al., 2021) | Qualitative case study, n=9 | Spain, an urban school for adults, 7 women with a low level of education. |
| 41 | (Serradell et al., 2020) | Qualitative case study, n=107 | Spain, an elementary school in a low SES urban area. 31 Moroccan immigrant mothers. |
| 42 | (Serrano-Alfonso et al., 2018) | Mix-method case studies, n=165 (quantitative); n=108 (qualitative) | Spain, 8 elementary schools in low SES urban areas. Non-academic mothers. |
| 43 | (Soler et al., 2019) | Longitudinal case study, n=53 | Colombia, a rural primary school, 19 students' relatives, and most of them mothers. |
| 44 | (Sordé Martí et al., 2012) | Qualitative study, n=40 | Spain, grassroots Roma women's movement. 30 Romani girls. |
| 45 | (Sordé et al., 2014) | Narratives, n=19 | Portugal, Romania, Spain, USA. Roma women and Roma immigrant women. |
| 46 | (Valls, 2014) | Literature and documentary review | Spain. Non-academic women. |
