# Supplementary material for: A systematic literature review and case study on the social impact of the other women’s contributions to education and dialogic feminism
Source: Front Sociol. 2024 Dec 9;9:1477983. doi: 10.3389/fsoc.2024.1477983 (PMC11664246; doi:10.3389/fsoc.2024.1477983)
Supplement: Supplementary file 2 [file Table_2.docx]

Supplementary Material

# Supplementary Table S2

***Social impact of educational actions to which the “other women” have contributed***

| **Action** | **Impact on themself** | **Impact on their families** | **Impact on their communities** | **Contributions to feminism** |
| --- | --- | --- | --- | --- |
| Dialogic spaces (Roma Women Students’ Gathering, Women Groups, Mutual Support Groups) | Creating new educational projects (Aiello et al. 2019) | Generating positive expectations to their children education (Aiello et al. 2019) | Promoting solidarity (Aiello et al. 2022; Sordé Martí et al. 2012; Sordé et al. 2014; Pulido et al. 2014; Ruiz-Eugenio 2016) | Dialogic Feminism (Puigvert and Elboj 2004; Arrufat 2004; Garcia Yeste, Ferrada, and Ruiz 2011; Ramis, Martín, and Íñiguez 2014; Valls 2014; Ruiz-Eugenio, Tellado, et al. 2023) |
|  |  | Roma women supporting their children with the online school tasks during lockdown (Aiello et al. 2022). | Becoming leaders in their communities (Aiello et al. 2019) | Roma feminism (Sordé et al. 2014; Aiello-Cabrera et al. 2024; Sordé Martí et al. 2012) |
|  |  |  | Creating and strengthening the social network (Puigvert and Elboj 2004) |  |
| Dialogic Gatherings (Literature, Science, Mathematics) | Improving self-confidence in their own academic, cultural and communicative abilities (de Botton et al. 2014; Duque 2015; Garcia Yeste et al. 2017; A. Flecha 2015) | Increasing learning and reading interactions at home (de Botton et al. 2014) | Overcoming the prejudice that people without an academic background cannot enjoy universal literature (Ruiz-Eugenio, Soler-Gallart, et al. 2023; A. Flecha 2015) | From adult learner to community activist for all women's right to education (A. Flecha 2015) |
|  | Improving language skills (de Botton et al. 2014) | Increasing their children reading and learning motivation (de Botton et al. 2014) | Overcoming racist prejudices about Muslim immigrant women (Ruiz-Eugenio, Soler-Gallart, et al. 2023) |  |
|  | Improving mathematics skills (Díez-Palomar 2020) | Use of scientific knowledge in decision-making on their family’s health-related habits (Buslón et al. 2020) | Creating a supportive environment (Ruiz-Eugenio, Soler-Gallart, et al. 2023; Ruiz-Eugenio et al. 2021) |  |
|  | Raising awareness about sciences behind social impact (Ruiz-Eugenio, Munte-Pascual, et al. 2023) |  | Creating and strengthening the social network (Garcia Yeste et al. 2017) |  |
|  | Overcoming the difficulty to access scientific knowledge (Buslón et al. 2020) |  | Promoting solidarity and an active citizenship facing the challenges of today’s society (Buslón et al. 2020) |  |
|  | Fostering analytical and critical thinking based on scientific evidence (Buslón et al. 2020) |  |  |  |
|  | Use of scientific knowledge in decision-making on their health-related habits (Buslón et al. 2020) |  |  |  |
| Dialogic model of conflict prevention and resolution | Mothers who did not participate before are involved in the dialogue spaces for consensus and monitoring the rules of coexistence in their children's school (Serradell et al. 2020; Oliver, Soler, and Flecha 2009) | The dialogue about the rules of coexistence at school is transferred to home and reinforces the children's adherence to them (Serradell et al. 2020; Oliver, Soler, and Flecha 2009) | Overcoming violence and gender violence in schools and community (Serradell et al. 2020; Oliver, Soler, and Flecha 2009) | Educators, teachers and the women of the community (mothers, older sisters, aunts, grandmothers, friends) jointly elaborate actions for the prevention of gender violence at school (Oliver, Soler, and Flecha 2009) |
|  |  |  | Promoting solidarity (Serradell et al. 2020) |  |
|  |  |  | Improving the school and community climate (Serradell et al. 2020) |  |
| Family education (literacy, writing and reading programs, etc.) & educative participation (Interactive Groups, mixed committees) | Improving self-confident to help their children with homework (A. Flecha 2012) | Improving their children motivation for learning (A. Flecha 2012; R. Flecha and Soler 2013; Garcia Yeste, Larena, and Miró 2012; Renta Davids, Aubert, and Tierno García 2018; Rodríguez-Oramas et al. 2022; Christou and Puigvert 2011) | From folkloric to intellectual contributors (Díez, Gatt, and Racionero 2011) | Demonopolisation of the expert knowledge (Melgar et al. 2011) |
|  | Increasing academic and linguistic skills (Girbés-Peco, Gairal-Casadó, and Torrego-Egido 2019; A. Flecha 2012; Ocampo-Castillo et al. 2023) | School absenteeism reduction (A. Flecha 2012; R. Flecha and Soler 2013; Rodríguez-Oramas et al. 2022; Soler et al. 2019) | Creating informal spaces for dialogue and participation (Díez, Gatt, and Racionero 2011) |  |
|  | Improving decision-making skills (Garcia-Carrion, Molina-Luque, and Molina-Roldan 2018) | Elimination of dropout in primary education (Garcia-Carrion, Molina-Luque, and Molina-Roldan 2018) | Involvement in decision-making spaces in the community (R. Flecha and Soler 2013; Girbés-Peco et al. 2020; Christou and Puigvert 2011) |  |
|  | Increasing autonomy (Girbés-Peco, Gairal-Casadó, and Torrego-Egido 2019) | Improving their children reading and mathematics outcomes (R. Flecha and Soler 2013; Renta Davids, Aubert, and Tierno García 2018; Rodríguez-Oramas et al. 2022) | Creating and reinforcing social networks and community support (Garcia Yeste, Morlà Folch, and Ionescu 2018; Girbés-Peco, Gairal-Casadó, and Torrego-Egido 2019; Ocampo-Castillo et al. 2023) |  |
|  | Self-esteem improvement (Garcia Yeste, Morlà Folch, and Ionescu 2018; Girbés-Peco, Gairal-Casadó, and Torrego-Egido 2019; Ocampo-Castillo et al. 2023) | Transforming classroom interactions (R. Flecha and Soler 2013; Christou and Puigvert 2011) | Overcoming stereotypical beliefs about the skills of women without academic education, immigrant women, or those from cultural minorities (Garcia Yeste, Larena, and Miró 2012; Christou and Puigvert 2011) |  |
|  | Increasing mothers and grandmothers involvement in school (Garcia Yeste, Morlà Folch, and Ionescu 2018; Ocampo-Castillo et al. 2023) | Improving children's behaviour at school (Renta Davids, Aubert, and Tierno García 2018; Rodríguez-Oramas et al. 2022) | Creating mutually supportive relationships between volunteers and families and between families themselves (Garcia Yeste, Ruiz Eugenio, and Comas 2019) |  |
|  | Increasing learning motivation and expectations for themselves and their children’s education (Garcia Yeste, Ruiz Eugenio, and Comas 2019; Garcia Yeste, Morlà Folch, and Ionescu 2018; Gómez-González, Tierno-García, and Girbés-Peco 2024) | Increasing learning interactions at home (Renta Davids, Aubert, and Tierno García 2018; A. Flecha 2012; Garcia Yeste, Morlà Folch, and Ionescu 2018) | Supportive relationships built on trust among the school’s staff and families (Khalfaoui, Garcia-Carrion, and Villardon-Gallego 2020) |  |
|  |  | Increasing children's participation in extended learning time activities (Renta Davids, Aubert, and Tierno García 2018) | Improving the school and community climate (R. Flecha and Soler 2013; Rodríguez-Oramas et al. 2022; Christou and Puigvert 2011) |  |
|  |  | Improving children’s health and health-related family habits inviting families to participate in the health education activities through egalitarian dialogue (A. Flecha, García, and Rudd 2011; Ruiz-Eugenio 2016) | Creating supportive environments through health literacy addressed to the expressed needs of the community (A. Flecha, García, and Rudd 2011) |  |
|  |  |  | Better prevention and early detection of drug abuse by taking into account the families' cultural intelligence (their knowledge and experience) (A. Flecha, García, and Rudd 2011) |  |
| Extending the learning time (tutorized library, support their daughters in continuing their education.) | Overcoming school drop-out rates and access to post-compulsory and university studies (Ruiz-Eugenio 2016; Munté Pascual et al. 2020) | Improving children’s learning and achievement (R. Flecha and Soler 2013; Rodríguez-Oramas et al. 2022) |  |  |
| Access to higher education | Networks of support among Roma girls encourage them to continue studying (Munté Pascual et al. 2020) | Expanding the educational opportunities of all Roma children (Munté Pascual et al. 2020). | The role of Roma women as key makers of change within the Roma community (Munté Pascual et al. 2020) | The dialogue between Roma women and non-Roma students contributes to overcome the stereotypes about the Roma culture, including their visions about the role of families in supporting education (Munté Pascual et al. 2020). |
|  |  |  |  | The transmission of the value of being a Roma woman and the struggle toward equality without rejecting the own culture has led to the interviewees to identify themselves as feminists (Munté Pascual et al. 2020) |
